# A two-stage RAG-LLM pipeline for roadmapping neuroscience research in operational environments

**DOI:** 10.3389/fnhum.2026.1884892

**Published:** 2026-10-14

**Authors:** Cara A. Spencer

**Affiliations:** Computer Science Department, University of Colorado Boulder, Boulder, CO, United States

**Keywords:** BCI-brain computer interface, biosensor, ecological validity, LLM, neuroimaging, retrieval-augmented generation (RAG)

## Abstract

**Introduction:**

Neuroscientific research and applications in real world environments are often constrained by limited access, equipment limitations, and high costs. Consequently, robustly selecting constructs to focus effort on is a high-impact task.

**Method:**

This study introduces a two-stage large language model (LLM) pipeline that generates research roadmaps from existing real-world data to inform research priorities in the field, given the state of the art. Stage 1 uses retrieval-augmented generation on operational logs to identify candidate psychological, social, and neurocognitive constructs and map their co-occurrence. Stage 2 evaluates how well these constructs are represented in the literature across four dimensions that commonly inform prioritization: ecological validity, neural correlates, biosensor protocols, and brain computer interface readiness, producing an evidence gap matrix. Pipeline evaluation combined human review, deterministic computations, and human-LLM alignment analysis.

**Results:**

Applying the pipeline to Mars Desert Research Station logs, Stage 1 identified 19 constructs. Stage 2 highlighted critical gaps in neuroimaging and measurement protocols, particularly for social domains, thereby identifying potential research priorities and risks.

**Discussion:**

This methodology offers a generalizable starting point for synthesizing operational logs into actionable research roadmaps, but the evaluation showed it still required human scrutiny.

## 1 Introduction

Nearly four decades ago, Winograd and Flores (1986) argued that understanding human thinking requires moving beyond purely descriptive accounts of task structure to account for the situated nature of human action. They emphasized that cognition is fundamentally embodied, context-dependent, and adaptive; consequently, they cautioned against static models disconnected from real-world practice. Since their seminal work, scholars have highlighted the persistent disconnect between research findings and practical applications, prompting numerous research agendas, traditions, and design approaches aimed at modeling neural processes in high-fidelity, ecologically valid environments (Andrade, 2018; Colusso et al., 2019; Cooke and Shope, 2017; Dalsgaard and Dindler, 2014; Hutchins, 1995). A recurring obstacle, however, is the gap between what neuroscience can measure in controlled laboratories and what is feasible to measure in operational settings (Hutchins, 1995). Costs, risks, and access limitations for many operational environments introduce significant challenges. Planning field research necessitates addressing several critical questions: (1) which psychological and cognitive constructs most urgently require neurophysiological monitoring; (2) whether these constructs have been studied under realistic conditions; and (3) the current limits of validated, practically deployable measurement protocols. Answering these questions comprehensively requires both a mechanism for identifying relevant constructs within a target environment and a systematic survey of the state of the art. In practice, both steps are resource-intensive and time-consuming. Generative artificial intelligence, specifically large language models (LLMs), has emerged as a scalable solution to support knowledge discovery in real-world neuroscience.

Field research in operational environments faces documented practical constraints. These settings are typically characterized by ill-structured problems, uncertain and dynamic conditions, shifting goals, action–feedback loops, time pressure, high stakes, and organizational constraints (Orasanu and Connolly, 1993). Examples include military deployments, Antarctic stations, emergency response operations, and long-duration spaceflight analogs. Access to research participants is challenged by organizational limitations, security requirements, or geographic remoteness. Consequently, sample sizes are small and increase slowly, reducing the statistical power needed to detect neurophysiological effects (Button et al., 2013). Participant burden is high, encompassing device use, data collection, and training on specialized equipment. These factors constrain the duration, complexity, and intrusiveness of measurement protocols. Furthermore, research timelines are governed by operational schedules rather than by experimental design preferences, and the cost of field data collection often exceeds sustainable research budgets (Stuster, 2010; Kanas and Manzey, 2008). These constraints collectively impede the development and testing of brain-computer interface (BCI) monitoring systems in real-world settings. Therefore, field research stakeholders may benefit from a new, principled, evidence-driven methodology to support construct prioritization.

Construct discovery and literature synthesis research that informs research planning and construct prioritization typically involves labor-intensive methods with automatable elements. For example, Ali et al. (2024) compared several natural language processing techniques for synthesizing literature into a literature review and found that LLMs performed this task well. The growing capability of LLMs for structured information extraction, conceptual clustering, and knowledge synthesis over large text collections offers a potential solution to the scalability problem in reviewing ever-growing literature (Thirunavukarasu et al., 2023; Brown et al., 2020). Systematic reviews of neuroimaging and BCI literature are time-intensive when conducted manually, yet the field cannot make principled research priority decisions without knowing the current state of evidence for each candidate construct. A carefully designed LLM pipeline, operating over literature retrieved from biomedical databases via structured API queries, could produce a first-pass evidence assessment across multiple constructs simultaneously. When combined with retrieval-augmented generation (RAG), which grounds LLM inference in retrieved passages from a specific corpus rather than relying on parametric knowledge alone, these systems can process large volumes of naturalistic operational text to surface recurring psychological themes to focus the literature synthesis (Lewis et al., 2020). This could compress a process that would otherwise require months of manual review into weeks or less. The scientific credibility of such a pipeline depends critically on how it manages the well-documented risks of LLM use in research contexts: hallucination, anchoring bias, and lack of transparency (Ji et al., 2023; Messeri and Crockett, 2024). Responsible use requires explicit validation procedures, retrieval grounding, human-in-the-loop checkpoints, and methodological transparency comparable to what is expected of any other analytic tool.

The current study demonstrates the use of RAG-LLMs for construct discovery from operational logs and for literature synthesis to prioritize constructs. We present a case study in which the pipeline is applied, along with an evaluation of the system. The pipeline has two stages: Stage 1: Construct Discovery and Stage 2: Literature and Gap Analysis. The results include a list of constructs extracted from the operational environment and a state-of-the-art summary of prior work on those constructs across four dimensions. These dimensions include: whether the construct has been studied in an operational context, whether neural correlates exist, whether biosensors or wearable devices can measure the construct, and whether a BCI protocol has been established in a real-world environment. Each of these dimensions is critical for determining which constructs are feasible to study in the field with neuroimaging techniques and which have readiness gaps. However, the methodology we describe is not specific to our case study or context: any domain where operational field logs, incident records, or structured written reports are available can serve as input to Stage 1, and the Stage 2 framework may generalize to any construct list and combination of scientific databases.

We ask the following questions: (1) What is the correspondence between what constructs field data reveal via RAG-LLM, via trained raters, and an expert taxonomy? (2) Is the RAG system accurate, relevant, comprehensive, and faithful? And (3) How well does the literature synthesis provide an accurate, relevant, comprehensive, and faithful summary of the state-of-the-art?

This study is organized as follows. Section 2 reviews the relevant literature on field research constraints in neuroergonomics, the construct selection problem, and LLM methods for scientific synthesis. Section 3 describes the MDRS log corpus and preprocessing decisions. Section 4 presents the pipeline methods, covering Stage 1 Construct Discovery and Stage 2 Literature and Gap Analysis. Section 5 presents the results from both stages. Section 6 discusses implications, limitations, and future work, followed by conclusions in Section 7.

## 2 Related work

### 2.1 Practical constraints of neuroergonomics research

Conducting neuroscience research in operational field environments carries difficulties that directly shape what is known and unknown about the neural underpinnings of performance in these settings. Access barriers are perhaps the most fundamental constraint: entry to military units, remote research stations, submarine crews, emergency services, and analog spaceflight facilities requires organizational approvals, security clearances, and logistical coordination that can extend timelines by years (Cooke and Shope, 2017). Once access is obtained, sample sizes are structurally limited by the small scale of the organizations and settings, and by the long duration of the missions under study. For example, spaceflight analogs typically collect data from small crews over multi-year data collection programs (Kanas and Manzey, 2008; De DeChurch et al., 2015). Operational datasets are typically supplemented by lab-based data collections or public datasets to help overcome this challenge, with the caveat that the ecological validity of the lab setting likely affects transferability (Andrade, 2018; Cheng and Phillips, 2014).

Participant burden and measurement noise are additional constraints to neuroergonomic measurement. High-density encephalography (EEG), high-density functional near-infrared spectroscopy (fNIRS), functional magnetic resonance imaging (fMRI), and other clinical neuroimaging protocols require specialized equipment, trained operators, controlled environmental conditions, and participant compliance with extended measurement sessions—all of which are difficult to reconcile with active operational settings (Mehta and Parasuraman, 2013; Scarapicchia et al., 2017). Even validated mobile neuroimaging systems, such as low-density fNIRS headsets and dry-electrode EEG, impose a meaningful burden on operators who are simultaneously trying to do their jobs (Di Flumeri et al., 2019; Pinti et al., 2018; Wascher et al., 2023). When neuroimaging is feasible, robust artifact and noise removal is essential because real-world tasks involve uncontrolled movement, lighting, and physiological confounds (Gwin et al., 2010; Scholkmann et al., 2014; Barker et al., 2016; Pinti et al., 2019; Harrivel and Hearn, 2012). The practical consequence is that neuroergonomic field studies are likely to be shaped by feasibility constraints: researchers preferentially study constructs that can be measured with relatively unobtrusive, robust, and field-deployable methods (Parasuraman, 2003; Diarra et al., 2025; Hamann et al., 2025). As a result, operationally relevant but difficult-to-measure constructs may be less visible in the empirical literature than constructs with established, low-burden measurement pipelines. Nevertheless, identifying relevant constructs and the corresponding state of the art, however limited it may be, informs construct selection.

These constraints motivate a methodological shift: before designing new data collection, the field should develop better tools to extract scientific value from existing datasets and use secondary analyses to generate hypotheses, refine constructs, and inform future operational study designs (Cheng and Phillips, 2014; Kelly et al., 2024). Crew or mission logs, personal journals, incident reports, and after-action reviews are routinely generated in many operational field environments as standard organizational practice. They frequently represent ecologically authentic records of operator experience. Additionally, they may be produced without research overhead and accumulate over time at no additional cost. The challenge is that extracting systematic, scientifically interpretable signal from these naturalistic text corpora at the scale required to build a credible construct inventory has historically required either intensive manual qualitative analysis or simple lexical methods that sacrifice nuance for scalability. LLM-based approaches offer a path between these extremes.

### 2.2 Construct selection for research prioritization

Across domains, a common approach to the research prioritization problem has been top-down: domain experts draw on behavioral health frameworks, clinical taxonomies, and prior literature to enumerate constructs of interest, which then drive experimental design and technology development (Cuthbert and Insel, 2013; Kotov et al., 2017; Mummah et al., 2016; Nilsen, 2015). Top-down taxonomies are valuable in that they provide much-needed theoretical foundations by encoding accumulated domain knowledge—especially when research is difficult to do in the actual context it is being applied to. Anchoring only in prior research, however, may mean these approaches underrepresent constructs that are context-specific, expressed in operators' own naturalistic vocabulary, or not yet prominent in the literature. A field operator who writes repeatedly about the particular cognitive burden of maintaining interpersonal cohesion in a tiny isolated group may be describing phenomena that sit between existing construct categories. Yet, these may be among the most mission-critical states to monitor.

Given the limitations of top-down taxonomies, research prioritization approaches rarely rely on those methods alone; instead, they balance them by incorporating data-driven insights using a variety of methods (e.g., NASA HRP HFBP Evidence Book). For example, unsupervised machine learning has been used to identify novel symptom clusters, and participatory design has been employed to ensure a clinical intervention addresses participants' priorities in a real-world context (Borsboom and Cramer, 2013; Kung et al., 2022; Lyon and Koerner, 2016). Data collection for these investigations is often restricted to a single or a few modalities due to the practical constraints described above. This is becoming less of a limitation with rapid advances in wearable sensors that have increased the data streams one device can measure. These devices alone, however, still face several challenges, including accuracy, stability, and robust diagnosticity (Kim et al., 2019). Optimizing the quality and quantity of signals extracted from individual devices (i.e., measurement economy) for biosensor protocols and BCI is therefore a very active area of research. A larger measurement economy can serve to alleviate some of the operational challenges of construct selection.

Traditional qualitative methods that similarly prioritize data-driven discovery and user-centered requirements to define constructs also allow the target to emerge from data or lived experience. Grounded Theory (Charmaz, 2006) and thematic analysis have been successful because some field environments more readily produce annotatable data, such as the diaries collected by Stuster (1996). Inductive and hybrid qualitative methods are commonly used because purely theory-driven coding can constrain what is noticed in the data (Braun and Clarke, 2006; Fereday and Muir-Cochrane, 2006; Linneberg and Korsgaard, 2019). Cognitive task and work analyses have also been a rich source of information regarding specific activities and the cognitive, social, and organizational constructs that support their completion within the broader socio-technical context (Rasmussen et al., 1994; Schraagen et al., 2000; Vicente, 1999; Crandall et al., 2006; Naikar, 2017). This class of methods facilitates construct discovery and description but depends on interviews or participants producing artifacts. The bridge from qualitative findings to neuroergonomics is less established, but the former's historical success in real-world settings may indicate its potential as a robust mechanism for informing construct selection for neuroergonomic investigations.

In the few cases where the field environment supports multiple modalities, such as spaceflight analogs that deploy behavioral, physiological, and self-report measures, inference is still constrained by small samples and environment-specific differences (De la Torre et al., 2024; Hill and Caldwell, 2022; Van Ombergen et al., 2017). Findings from more controlled or more replicable studies can therefore complement field collections and inform measurement selection, hypotheses, and model development, but their transferability depends on the fidelity of the laboratory or analog context (De la Torre et al., 2024; Schlosser et al., 2025). Taken together, these methods provide the highly needed field-derived data to support the top-down foundations for prioritizing which constructs future research should investigate.

### 2.3 LLMs and RAG for literature synthesis

Large language models can perform tasks relevant to systematic scientific review: abstract screening, evidence extraction, PICO element identification, and structured summarization of research findings across multiple documents (Agrawal et al., 2022; Kusa et al., 2023). Several studies have benchmarked LLM performance on these tasks against human reviewers, finding accuracy approaching human levels for structured extraction tasks while identifying characteristic failure modes including hallucination, anchoring on training data distributions, and difficulty with tasks requiring deep domain inference that require mitigation in research contexts (Thirunavukarasu et al., 2023; Ji et al., 2023). Consequently, the field is increasingly developing methodological best practices for responsible LLM use in science, emphasizing prompt-design transparency, retrieval grounding, output validation against authoritative sources, and reporting uncertainty (Shah and Bender, 2022; Messeri and Crockett, 2024).

Hallucination, the generation of plausible but factually unsupported content, is the most consequential failure mode for literature synthesis applications. An LLM performing gap analysis may cite a non-existent study, misattribute a finding to the wrong source, or produce an evidence summary that reflects training data priors rather than the actual literature retrieved for the task. These risks are well-documented and motivate the use of (1) retrieval grounding, which constrains model outputs to the retrieved documents; (2) citation verification, which allows any cited document to be cross-referenced against the retrieval database; and (3) human review of a sampled fraction of outputs (Guu et al., 2020; Ji et al., 2023). The pipeline presented in this study implements all three mitigations and reports inter-rater agreement statistics for human validation, establishing the empirical basis for confidence in the evidence gap matrix outputs.

RAG extends LLM inference by retrieving relevant passages from an external vector-indexed knowledge base and prepending them to the model's context window before generation (Lewis et al., 2020). This approach substantially improves factual grounding on knowledge-intensive tasks, enables processing of document collections far larger than any model's context window permits directly, and—critically for scientific applications—produces outputs that can be traced back to specific retrieved passages for verification (Guu et al., 2020; Arslan et al., 2024). In scientific and clinical knowledge extraction contexts, RAG systems have demonstrated improved performance on structured information extraction from research databases, clinical note analysis, and domain-specific question answering over proprietary document collections (Han et al., 2024; Yang et al., 2025; Siriwardhana et al., 2023).

For construct discovery from operational text corpora, RAG offers three specific advantages. First, grounding in retrieved passages enables *post-hoc* verification of each construct identification against the supporting source text. This supports the interpretability requirements of responsible research use. Second, chunking and retrieval strategies can be calibrated to the corpus's semantic structure, ensuring that contextually coherent units (e.g., complete reflective passages rather than arbitrary text windows) form the basis of LLM inference. Third, the auditable retrieval log recording which passages were retrieved for each inference call constitutes a methodological artifact that can be inspected, reproduced, and used to characterize the empirical basis of the construct inventory. These properties make RAG substantially more suitable for construct discovery in scientific research contexts than unconstrained LLM prompting.

### 2.4 Spaceflight neuroergonomics as a pipeline demonstration context

More specific to this work, construct prioritization within spaceflight neuroergonomics is transitioning to an increasingly data-driven approach to complement the top-down models inherited from clinical and occupational health, and industrial and organizational psychology. The reports in the NASA Human Research Program (HRP) Human Factors and Behavioral Performance (HFBP) Evidence represent the current gold standard, synthesizing risks from existing spaceflight and spaceflight analog research (National Aeronautics and Space Administration Human Research Program, 2025). This taxonomy also incorporates standards and occupational metrics originally developed for analogous populations, such as military and healthcare workers. The unique phenomena associated with the isolated, confined, and extreme (ICE) environment of spaceflight, however, are still being characterized. Research already underway to bridge this gap is reflected by the shift toward ecologically valid methods that prioritize the lived experience of spaceflight crews (Bell et al., 2026). The current study contributes to these efforts by using qualitative methods on decades of raw field logs at a spaceflight analog.

The methodological case for bottom-up, data-driven construct discovery has been articulated within qualitative research traditions. Grounded Theory, in particular, explicitly advocates allowing theoretical constructs to emerge from systematic engagement with primary data rather than being imposed from pre-existing frameworks (Charmaz, 2006; Glaser and Strauss, 1967). Applied to field log data, a grounded approach would allow the constructs most saliently represented in crew experience to inform the scientific agenda. Prior qualitative analyses of ICE behavioral data have produced construct inventories through diary analysis and ethnographic observation (Stuster, 1996; Kanas and Manzey, 2008), but these studies are labor-intensive and constrained by participation and the volume of text they can systematically analyze. A computational implementation of the inductive logic underlying Grounded Theory that can process thousands of logs represents a meaningful advance in the methodological toolkit available for construct discovery.

ICE environments (e.g., Antarctic overwintering stations, submarine deployments, polar expeditions, and spaceflight analog facilities) provide a well-characterized research context in which the constraints described above manifest in particularly concentrated form (De la Torre et al., 2024; Lugg, 2005; Stuster, 2010). Long-duration missions in these environments produce documented psychological effects, including motivational decline, escalation of interpersonal conflict, affective trajectory shifts associated with mission phase, and cognitive performance decrements attributable to sustained stress and sleep disruption (Schorn and Roma, 2020; Schlosser et al., 2025; Kanas and Manzey, 2008; Stuster et al., 2018). Understanding where breakdowns occur, whether interpersonally, individually, or within human-machine systems in these environments, has direct relevance to mission safety and performance. This motivates sustained interest in developing neurophysiological monitoring and BCI approaches that can detect emerging behavioral health risks without relying solely on self-report (Strangman et al., 2014; Kanas and Manzey, 2008).

The Mars Desert Research Station (MDRS) log archive provides the specific demonstration corpus for the presented RAG-LLM pipeline. MDRS crews conduct one- to multi-week simulated Mars missions in a Utah desert habitat. They operate under conditions such as physical confinement, communication constraints, small fixed teams, resource limitations, and extravehicular activity schedules, which together produce the core ICE stressor profile on an accessible, long-running research platform (Clancey, 2004). The MDRS log archive spans more than two decades of continuous operation. It includes structured commander reports, journalist entries, and personal reflective logs submitted across hundreds of missions, providing a volume and longitudinal range of naturalistic operational text that is unusual in the ICE research literature (Stuster et al., 2018). Importantly, these logs were not designed for research—they are authentic operational records. This makes them well-suited to a bottom-up analysis intended to surface constructs that pre-structured research instruments might miss. Further, this research site has a history of neuroergonomic and BCI research, meaning the constructs derived from this pipeline could realistically be applied to future research agendas at MDRS.

## 3 Materials: MDRS crew log corpus

### 3.1 Corpus description

The MDRS operators maintain a publicly accessible archive of crew logs submitted during analog missions conducted at its Utah desert facility. For this study, we utilized 2,521 log documents spanning 2001 to 2026, covering over 300 discrete missions. Each mission had between three and eight crew members. The logs were fetched from the MDRS reports website using the WordPress REST API. The corpus encompasses all logs from five primary log types. Sol Summaries are highly structured and include daily activities, a look-ahead plan, anomalies, mission status, and crew health status (*n* = 1, 088). Commander Reports are operational summaries authored by mission commanders that document more informally the crew activities, technical issues, and other mission details (*n* = 181). Journalist Reports are similar daily narrative entries, but authored by a designated crew journalist and emphasizing the human and experiential dimensions of mission life (*n* = 978). Mission Summaries were typically written by the commander(s) as a single document covering the mission's higher-level themes and activities at its conclusion (*n* = 94). Lastly, the Science Reports included documentation of the research being done at the station by that crew, as well as reflective, first-hand accounts of the research and operational experience (*n* = 180). Combined, the corpus comprises approximately 1 million words of crew-authored naturalistic text.

### 3.2 Pre-processing decisions

Preprocessing was guided by the goal of retaining psychologically and cognitively reflective content while removing material that is primarily logistical, technical, or administratively formulaic. Selecting five log types from the thirteen available was the primary mechanism for enforcing this scope. The chosen categories (i.e., Sol Summaries, Commander Reports, Journalist Reports, Mission Summaries, and Science Reports) are those most likely to contain first-person reflective content, while excluded categories such as engineering reports and HSO reports are dominated by equipment status, supply inventory, and operational checklists. Within the retained log types, no further regex-based section filtering was applied, with the trade-off that some logistical content (e.g., daily activity timestamps in commander reports) reaches the embedding store and contributes to the candidate chunk pool. No content was excluded based on valence or sentiment.

Each fetched log was saved as a UTF-8 plain-text file in a per-category subdirectory under the corpus root, with a five-line header recording the report type, mission identifier (parsed from the post title or content via a regex matching common “Crew NNN” / “MDRS NNN” patterns), date, title, and source URL, followed by the report body. A manifest file tracked the slug, post identifier, mission, date, category, title, and file path of every retrieved log, supporting incremental re-fetching and downstream auditing. We note that the per-document metadata captured in these headers is not currently propagated into the chunk-level metadata stored in the vector index, which retains only the source-document identifier and the chunk's starting character offset. A human reviewer audited a sample of 30 log entries (six from each log type) and confirmed authentic crew authorship in 100% of sampled entries, providing a first validation step for the corpus.

## 4 Method

Broadly, the RAG-LLM system has two stages: Stage 1: Construct Discovery followed by Stage 2: Literature and Gap Analysis, described in detail in the following sections and depicted in Figure 1.

**Figure 1:**
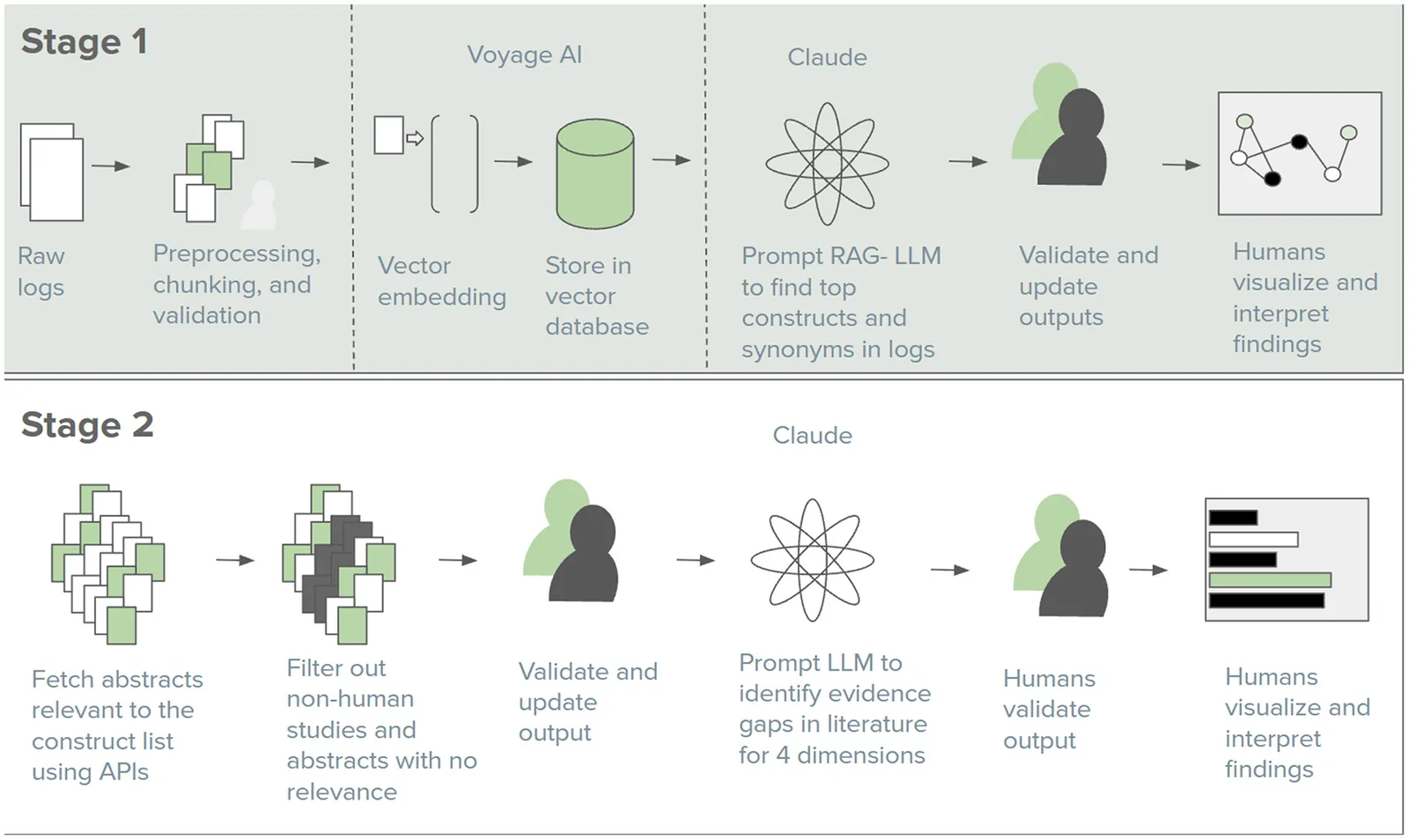
Description of the RAG-LLM system from pre-processing to Stage 1 Construct Discovery to Stage 2 Literature and Gap Analysis.

### 4.1 Stage 1 methods: construct discovery

#### 4.1.1 RAG architecture

The Stage 1 Construct Discovery pipeline was implemented as a retrieval-augmented generation (RAG) system with three components: a preprocessing and chunking module, a dense embedding and vector-indexing module, and an LLM inference module. After preprocessing, the log corpus was segmented into overlapping character-windowed chunks of 600 characters with a 100-character overlap (i.e., a 500-character stride), and any residual fragment shorter than 150 characters was discarded. Character-windowed chunking, rather than tokenizer-aligned or sentence-aligned chunking, was chosen because crew log entries vary widely in punctuation, paragraphing, and orthographic conventions (including code-mixing, informal abbreviations, and non-native English authors). A fixed character window provides uniform retrieval granularity independent of tokenizer choice or downstream embedding-model assumptions. Each chunk was encoded using the voyage-3-large embedding model and persisted to a ChromaDB index configured for cosine similarity, with the chunk's source-document identifier and starting character offset retained as metadata for downstream attribution.

The corpus was swept using a fixed set of eight broad seed queries (full text is provided in Supplementary material), chosen *a priori* to span the breadth of crew-experience domains anticipated to appear in MDRS logs. The prompts covered team dynamics and morale, stress and cognitive workload, interpersonal conflict, isolation and monotony, decision-making under uncertainty, emotional regulation, autonomy and mission purpose, sleep, and physical and cognitive interfaces. For each seed query, the top 20 chunks were retrieved by cosine similarity in the embedding space, and the union of retrieved chunks across the eight sweeps was deduplicated by chunk identifier to form the candidate pool for construct extraction. We then performed construct extraction at the per-chunk level. Each chunk in the deduplicated pool was independently submitted to the LLM with the construct-extraction prompt described below, and extracted constructs were aggregated across chunks. Claude-sonnet-4-6 was selected for its adequate performance and low cost. This decoupling of retrieval from inference, rather than performing per-call top-*k* retrieval against a per-construct query, was deliberate. Because the construct vocabulary is itself the target of discovery, query construction at inference time would be circular. Sweeping with deliberately broad seeds and then iterating extraction across all retrieved chunks places the LLM in the role of detector rather than confirmer, producing a candidate construct list whose composition is determined by the corpus rather than by query design.

#### 4.1.2 LLM prompting strategy for construct extraction

We framed construct extraction as a structured information extraction task. Each inference call presented the LLM with a set of retrieved log passages and instructed it to: (1) identify any psychological, emotional, interpersonal, or cognitive states described or implied in the passages; (2) express each identified state as a candidate construct label with a brief operational definition grounded in the passage content; (3) provide the specific passage excerpt or excerpts that most directly evidence the construct; and (4) assign a confidence rating (low/medium/high) based on how explicitly or inferentially the construct is supported by the text. Per RAG guidelines, system prompts explicitly instructed the model to ground all construct identifications in the provided passages, avoid importing construct labels from external psychological frameworks without corresponding textual support, and flag constructs that relied on inference beyond what the passages directly supported. All prompts are included in Supplementary material.

#### 4.1.3 Creating the construct inventory

Repeated queries using broad retrieval seeds across the full corpus produced a raw list of candidate constructs with substantial terminological redundancy (e.g., “exhaustion,” “fatigue,” and “tiredness” returned as distinct labels by different inference calls). Canonicalization proceeded in three steps. First, a frequency filter retained only labels (matched case-insensitively) appearing in at least three independent extraction events, suppressing one-off labels arising from idiosyncratic LLM phrasings and ensuring that admitted constructs were supported by multiple distinct passages. Second, the filtered candidate list, together with each candidate's frequency, operational definition, and supporting chunk identifiers, was submitted to Claude-opus-4-7 in a single canonicalization call. The model was instructed to group synonymous and near-synonymous candidates under canonical labels while preserving meaningful distinctions between related but non-identical constructs (e.g., “Crew Cohesion” vs. “Crew Morale”). Single-call LLM canonicalization was chosen over classical embedding-distance clustering because canonicalization in this domain requires reasoning about operational meaning rather than surface lexical similarity. The post-filter candidate list was small enough to fit within a single Opus context window. Third, the resulting canonical inventory was independently reviewed by two human raters, who assessed whether the LLM's groupings were semantically appropriate, whether any canonical clusters warranted subdivision, and whether each operational definition adequately represented the construct as evidenced by the supporting log excerpts.

Because LLM queries are non-deterministic, we ran the prior steps 30 times to generate a construct inventory and assess selection stability. The Stage 1 pipeline contains three stochastic components: the per-chunk construct extraction issued to Claude Sonnet, the single canonicalization-and-merging call issued to Claude Opus, and the synonym-expansion call. Of these, the canonicalization call is the dominant source of run-to-run variation in the final construct inventory. Per-chunk extraction is also stochastic, but its variance is averaged across hundreds of calls, and the raw construct pool is correspondingly stable. Therefore, we included only a stability-selection module that resamples the canonicalization step.

Following the stability-selection framework of Meinshausen and Bühlmann (2010) and the consensus-clustering approach of Monti et al. (2003), as adapted to topic-model outputs by Greene et al. (2014), the canonicalization call was executed *N* = 30 times against the same filtered raw-construct pool. We chose *N* = 30 so the standard error on a binary selection probability would not exceed 0.09; pilot runs at *N* = 5 produced 95% Wilson intervals on selection probabilities that were too wide to support principled inclusion decisions.

All canonical constructs from the 30 replications were pooled, embedded as the concatenation “〈 label〉: 〈 definition〉” using voyage-3-large, and then meta-clustered with average-linkage agglomerative clustering on cosine distance, without a fixed cluster count and with a distance threshold of 0.10. The threshold was set by inspecting borderline pairs and corresponds to the natural gap between semantic near-synonyms (e.g., “crew cohesion”/“team cohesion”, cosine distance ≈ 0.10) and genuinely distinct constructs (≥ 0.40). See Supplementary material for the plot of the pairwise cosine distance distribution across pooled constructs. Each resulting meta-cluster represents one putative canonical construct.

For each meta-cluster, we computed a selection probability equal to the proportion of distinct runs contributing at least one member, together with a Wilson 95% confidence interval. The final stable Stage 1 inventory comprises every meta-cluster whose Wilson lower bound exceeded 0.50, i.e., constructs whose long-run appearance rate is statistically distinguishable from a minority of runs. We retained no meta-clusters below this threshold as marginal constructs. Within each retained meta-cluster, the representative label was assigned by run-mode of the canonical labels. Two scalar diagnostics characterize pipeline stability at the global level: the mean pairwise Jaccard similarity of meta-cluster ID sets across the 302 run pairs, and the mean per-cluster selection probability across the stable inventory. Both are reported in Section 5. A list of verified synonyms was retained for Stage 1 validation and searching databases in Stage 2 and is provided in Supplementary material.

#### 4.1.4 Validation

To first demonstrate that the Stage 1 construct inventory reflects genuine patterns in the MDRS corpus rather than artifacts of LLM parametric knowledge about psychological constructs, we reviewed the quality of the canonicalization and conducted a two-step systematic comparison between the validated construct list, trained rater construct extraction, and the NASA HRP HFBP Evidence reports. This effort also addressed the contrastive question motivating Stage 1: What is the correspondence between what constructs field data reveal via RAG-LLM, trained raters, and an expert taxonomy?

For each Stage 1 construct, two trained raters independently reviewed a random sample of 40 logs (eight from each log category). They extracted salient constructs using Grounded Theory methods after achieving inter-rater reliability of κ = 0.76. They then reached consensus on a final inventory of canonical construct labels. Afterward, they assessed the final inventory of LLM-discovered constructs' quality and correspondence to their inventory and to the NASA HRP HFBP Evidence reports using keyword searches and the synonyms identified in Step 4.1.3. Each canonical group was reviewed for coherence and uniqueness by two raters, with an inter-rater reliability of κ = 0.96. Taxonomy correspondence was scored on a three-point scale: *direct correspondence* (the construct maps clearly to a construct in the raters' inventory or evidence report), *partial correspondence* (the construct conceptually overlaps with a construct in the raters' inventory or evidence report but is not an exact match), or *no correspondence* (the construct does not map to any in the raters' inventory or evidence reports). Inter-rater reliability for this task was also assessed using Cohen's kappa, yielding κ = 0.73. These results are presented in Table 1. Additionally, following the Wallat et al. (2024) framework for evaluating LLMs using RAG, we assessed RAG comprehensiveness, relevance, attribution correctness, and faithfulness to the prompt. The raters completed this evaluation using a random sample of 100 logs and the RAG prompt used for each construct.

**Table 1:** Stage 1 construct inventory (*N* = 19) and passage frequency across the MDRS log corpus.

Rank	Construct	Passage frequency	NASA HRP HFBP correspondence	Trained Rater correspondence
1	Crew cohesion	26	T	T
2	Emotion management/regulation	17	T	T
3	Sleep quality/disruption	14	T	T
4	Cognitive fatigue/performance degradation	11	T	T
5	Crew morale	10	T	P
6	Autonomy	9	T	T
7	Acute stress response	7	T	N
8	Self-awareness	6	P	P
9	Interpersonal trust	6	T	T
10	Mutual support orientation	6	T	T
11	Agentic leadership motivation	6	T	T
12	Goal-directed motivation	5	P	P
13	Boredom	5	P	P
14	Privacy deprivation	5	T	T
15	Behavioral adaptation	4	T	P
16	Fatigue	4	T	N
17	Individualistic competitive orientation	3	T	N
18	Isolation stress	3	T	T
19	Other-oriented emotional concern	3	T	T

T, Total Correspondence; P, Partial Correspondence; N, No Correspondence.

#### 4.1.5 Visual output: construct relationship

After validation, we represented the construct inventory as an undirected graph to visualize the structure of the operational construct space and identify potential measurement economies (i.e., cases where constructs that crew members describe together in the same passages might be captured by overlapping monitoring channels). Nodes represent the validated canonical constructs, with node size scaled to passage frequency (the number of distinct retrieved log chunks in which the construct was evidenced). Edges connect any two canonical constructs that co-occur within the same retrieved log chunk in at least two distinct chunks; edge weight is the count of distinct chunks in which the pair co-occurs. The minimum-co-occurrence threshold filters out one-off pairings that arise from a single chunk and do not reflect a stable relational pattern in the corpus.

The graph was constructed by mapping each raw extraction event to its parent canonical construct and grouping the resulting canonical labels by source chunk. Co-occurrence is operationalized at the chunk level because the chunk is the unit at which the LLM made independent extraction judgments; defining co-occurrence over coarser units (e.g., whole logs) would allow a single discursive contributor to drive spurious pairings, and no finer unit is available. The graph was rendered using NetworkX (Hagberg et al., 2008) with a Kamada–Kawai force-directed layout that positions more strongly connected constructs closer together while minimizing edge crossings.

### 4.2 Stage 2 methods: gap analysis

#### 4.2.1 Literature retrieval pipeline

For each construct in the validated Stage 1 output, structured literature queries were formulated and submitted to three bibliographic databases: PubMed (National Library of Medicine) via the E-utilities API, Semantic Scholar (Allen Institute for AI) via the Graph API, and IEEE through the Xplore Metadata API. These databases were selected for their expected coverage of the neuroergonomics, biomedical, and neuroengineering literature and free access. Construct labels were augmented before query construction to include the Stage 1-generated synonym list.

Retrieval was configured to be exhaustive on every source rather than capped at a per-construct budget since the design intent of this survey is to recover the complete match set. Because each API truncates a single response differently, exhaustiveness is realized through a source-specific pagination mechanism. PubMed uses the E-utilities history server (WebEnv/QueryKey) and pages the entire result set by retstart in batches of 200; the history server is required to page beyond the ~10, 000-record ceiling that plain retstart imposes. Semantic Scholar uses the bulk search endpoint (/paper/search/bulk), which token-paginates the complete match set rather than only the first 1, 000 results returned by the relevance endpoint. IEEE Xplore pages by start_record in steps of 200 up to the reported total_records.

*PubMed. Two query variants were issued per construct*. A broad variant combined the label-and-synonym title/abstract phrase clause with a neuro-modality clause (EEG, electroencephalograph*, fMRI, fNIRS, functional near-infrared, neuroimaging, neural correlate(s), brain, neural, cortical, hemodynamic, autonomic) together with the BCI vocabulary described below, restricted to human subjects (humans[MeSH Terms]) and English language publications. A second, BCI-targeted variant combined the same phrase clause with the BCI vocabulary alone (brain computer interface, brain-computer interface, brain-machine interface, BCI, BMI, neural interface, neurofeedback, neuroadaptive, passive BCI, closed-loop neurofeedback, neuroergonomic) and deliberately *omitted* the humans[MeSH Terms] gate. This omission is intentional: a substantial fraction of BCI and neuroengineering work is indexed in PubMed but not in MEDLINE and therefore carries no MeSH terms, so a [MeSH Terms] clause silently excludes it; non-human records are instead removed by the downstream species filter. Results from the two variants were pooled and de-duplicated by PMID, falling back to DOI and then to a normalized title.

*Semantic Scholar*. The bulk endpoint supports a compact boolean query syntax (| for disjunction, + for conjunction, and quoted phrases), so a single boolean query was issued per construct in place of the relevance-ranked fan out used previously. The query conjoined a disjunction of the label-and-synonym phrases with a disjunction of methodological and operational anchors: neuroimaging modalities (EEG, fMRI, fNIRS, near-infrared, neuroimaging), neural correlate, biosensor/wearable, the BCI vocabulary, and operational-setting terms (isolated confined extreme, spaceflight, Antarctic, submarine, analog). No *fieldsOfStudy* restriction was applied to avoid over restriction. Records without an abstract were discarded, and results were de-duplicated by paper identifier.

*IEEE Xplore*. Because IEEE Xplore is the principal venue for the BCI and neuroengineering literature, two complementary passes were issued per construct through the querytext field, which supports boolean AND/OR operators and quoted phrases. Each pass conjoined a disjunction of the label-and-synonym phrases with a methodological disjunction: a BCI pass (brain-computer interface, brain computer interface, brain-machine interface, BCI, neurofeedback, neuroadaptive, passive BCI, neural interface) and a neuro/biosensor pass (EEG, fNIRS, fMRI, electroencephalography, biosensor, wearable, neuroergonomics, physiological computing, neural correlate). Splitting the neural-interface and sensing/biosensor terminology across two passes, rather than combining them into one long disjunction, keeps each querytext within IEEE's parsing limits and ensures that neither family of terms is crowded out. For each returned record, the article title, abstract, publication year, publication title, DOI, and index terms (IEEE, author, and dynamic index terms) were retained; records without an abstract were discarded, and the two passes were pooled and deduplicated by DOI, falling back to the IEEE article number and then to a normalized title.

Across all three sources, only abstracts (and, for IEEE, index terms) were retrieved; full text was not obtained. Three deterministic post-retrieval filters were then applied to the pooled records. A *species filter* drops abstracts containing non-human species terms (e.g., zebrafish, mice, rats) when no human-subject term (“human,” “participant,” “patient,” “subject,” “volunteer”) is present. A *relevance filter* requires at least one token longer than three characters from the construct label or one of its synonyms to appear in the title or abstract. A *clinical and age exclusion filter* removes purely clinical population-based studies (i.e., pure clinical-epidemiology, questionnaire, or disease or injury treatment studies that would score zero on every evidence dimension), and studies looking at populations under 18 years old due to their irrelevance to operational environments where participants are typically over that age and healthy. Per construct retain and drop counts for all filters are reported in the Results. Filtering by publication type (e.g., excluding letters, editorials, and conference abstracts) was not applied. All queries were executed within a single retrieval window (June 2026), establishing a uniform evidence cutoff date. Two human raters examined a sample of 100 abstracts excluded by the deterministic filters to estimate the false-negative rate, achieving an inter-rater reliability of κ = 0.92.

#### 4.2.2 Gap dimensions and evidence rubric

Each retrieved abstract set was evaluated along four evidence dimensions that together characterize the translational pipeline from laboratory neuroscience to deployable field monitoring, which would reasonably factor into construct prioritization.

*Dimension 1—ICE Ecological Validity* assesses whether the construct has been studied under realistic field conditions or in comparably ecological high-fidelity settings, as opposed to controlled laboratory paradigms.

*Dimension 2—Neural Correlate Identified* assesses whether the construct has any published neurophysiological correlate (e.g., EEG signature, fMRI activation pattern, fNIRS hemodynamic response, autonomic marker, or other neural index) regardless of the ecological setting in which the correlate was established. This dimension separates “is the construct neurally tractable in principle” from “has it been tractably measured in the field,” allowing the evidence gap matrix to distinguish constructs with no strong neuroscientific basis established from constructs whose laboratory basis has not been operationalized for field use.

*Dimension 3—Validated Biosensor Protocol* assesses whether a specific construct has a validated, deployable biosensor/neuroimaging protocol existing outside a laboratory setting (direct evidence; protocols in a laboratory only were marked as indirect evidence).

*Dimension 4—BCI Implementation Ready* assesses whether the construct has been used as a control signal or target state in a working BCI system.

Evidence on each dimension was labeled 0/1/2 (none/indirect/direct) for each abstract after filtering, and the Evidence Gap Matrix cells contain the count of supporting abstracts. All evidence labels assigned by the LLM were required to be grounded in specific retrieved abstracts, with bibliographic information for every evidentiary claim recorded in the model's structured output and subsequently verified against the retrieval database. The Claude-opus-4-7 model was selected for its better-reported complex reasoning, verifiable outputs, and improved tokenizer.

#### 4.2.3 Hallucination mitigation and human validation

Complementary procedures were implemented to manage hallucination risk in Stage 2 and to evaluate the system. In addition to the prompt's explicit instruction to retrieve only from the citation database, every reference cited by the LLM in its evidence assessments was automatically cross-referenced against the per-construct retrieval database provided as the LLM's input context. Cross-referencing used a two-stage title-matching procedure: a normalized substring match (case-insensitive, alphanumeric-only, whitespace-collapsed) was attempted first, and citations that did not produce a substring match were checked against a SequenceMatcher similarity ratio above 0.85 against the retrieved-title set. References that matched a retrieved abstract were marked verified; those that did not were flagged as unverified and recorded in a per-construct citation audit. We report the aggregate flagging rate (unverified references/total cited references) as a pipeline diagnostic in Section 5. We note that this procedure detects only fabricated references that present as titles not in the LLM's input context; it does not detect cases in which the LLM cites a real retrieved paper but misrepresents its findings, which remain the target of the human validation steps below.

Second, two human reviewers examined the labeled abstract set for a powered sample of 200 which were dimension-stratified (CI half-widths ±0.099–0.123) and assigned an independent evidence label using the same rubric, blind to the LLM-assigned label. Cohen's kappa was computed to characterize the human–human agreement on the dimension labeling (ICE: κ = 0.89; Neural Correlate: κ = 0.95; Biosensor: κ = 0.82; BCI: κ = 0.91). Then human–LLM agreement was calculated and systematic discrepancies were used to identify failure modes in the prompting strategy, with iterative prompt revisions applied until agreement exceeded a pre-specified threshold for each dimension (κ≥0.65). We also completed a *post-hoc* review of all evidence gap matrix cells with a zero (i.e., indicating zero supporting abstracts), since this was an unexpected result. This human-in-the-loop design operationalizes the principle that LLM synthesis should function as a powerful but fallible first-pass tool, with domain-expert scrutiny serving as a necessary validation step before scientific conclusions are drawn.

#### 4.2.4 Evidence gap matrix construction

To summarize the state of the art at a high level, the Stage 2 output is a validated evidence gap matrix. The matrix is a construct-by-evidence-dimension table in which each row represents a validated Stage 1 construct and each column represents one of the four gap dimensions, with cell values reflecting the count of abstracts found. Constructs are ordered by ascending evidence count, with the largest gap at the top. Rows with low counts can be interpreted as mental states whose presence in authentic field text is documented (by virtue of having survived Stage 1 frequency filtering and expert canonicalization) and for which the existing literature is most underdeveloped.

## 5 Results

### 5.1 Stage 1 results: stability of the construct inventory

Across *N* = 30 successfully completed replications of the canonicalization step, the pipeline produced a mean of 18.75 canonical constructs per run (*SD* = 0.52). Pooling and meta-clustering were set at a cosine distance threshold of 0.10, selected from a stability plateau in which thresholds in [0.10, 0.12] produced the same 19-cluster partition. It yielded 19 meta-clusters, all of which met the Wilson lower-bound inclusion criterion (${\hat{\pi }}_{\text{lower}\;\text{CI}}\geq 0.50$) and constitute the final stable construct inventory used in Stage 1 validation and Stage 2. No meta-clusters fell below the inclusion threshold. The mean pairwise Jaccard similarity of meta-cluster ID sets across the 435 run-pair combinations was 0.976 (*SD* = 0.035), substantially above the ≈0.80 range that Greene et al. (2014) treat as indicative of a stable topic-discovery configuration. The mean per-cluster selection probability across the stable inventory was 0.987. A total of 16 of the 19 stable constructs appeared in 100% of completed runs. Three constructs, namely *Fatigue* ($\hat{\pi }=0.96$), *Isolation Stress* ($\hat{\pi }=0.93$), and *Other-Oriented Emotional Concern* ($\hat{\pi }=0.86$), appeared in 86–96% of runs, reflecting category-boundary ambiguity with their nearest stable neighbors (i.e., *Cognitive Fatigue/Performance Degradation, Sleep Quality/Disruption*, and *Individualistic Competitive Orientation*, respectively). The human reviewers additionally modified one label based on the evidence the LLM provided being broader than what the label indicated, namely: “Emotional Self-Awareness” was changed to “Self-Awareness.” Figure 2 plots the selection probabilities in descending order with associated Wilson intervals; the gap separating the two bands corresponds to constructs whose boundaries with neighboring constructs are themselves variable across runs.

**Figure 2:**
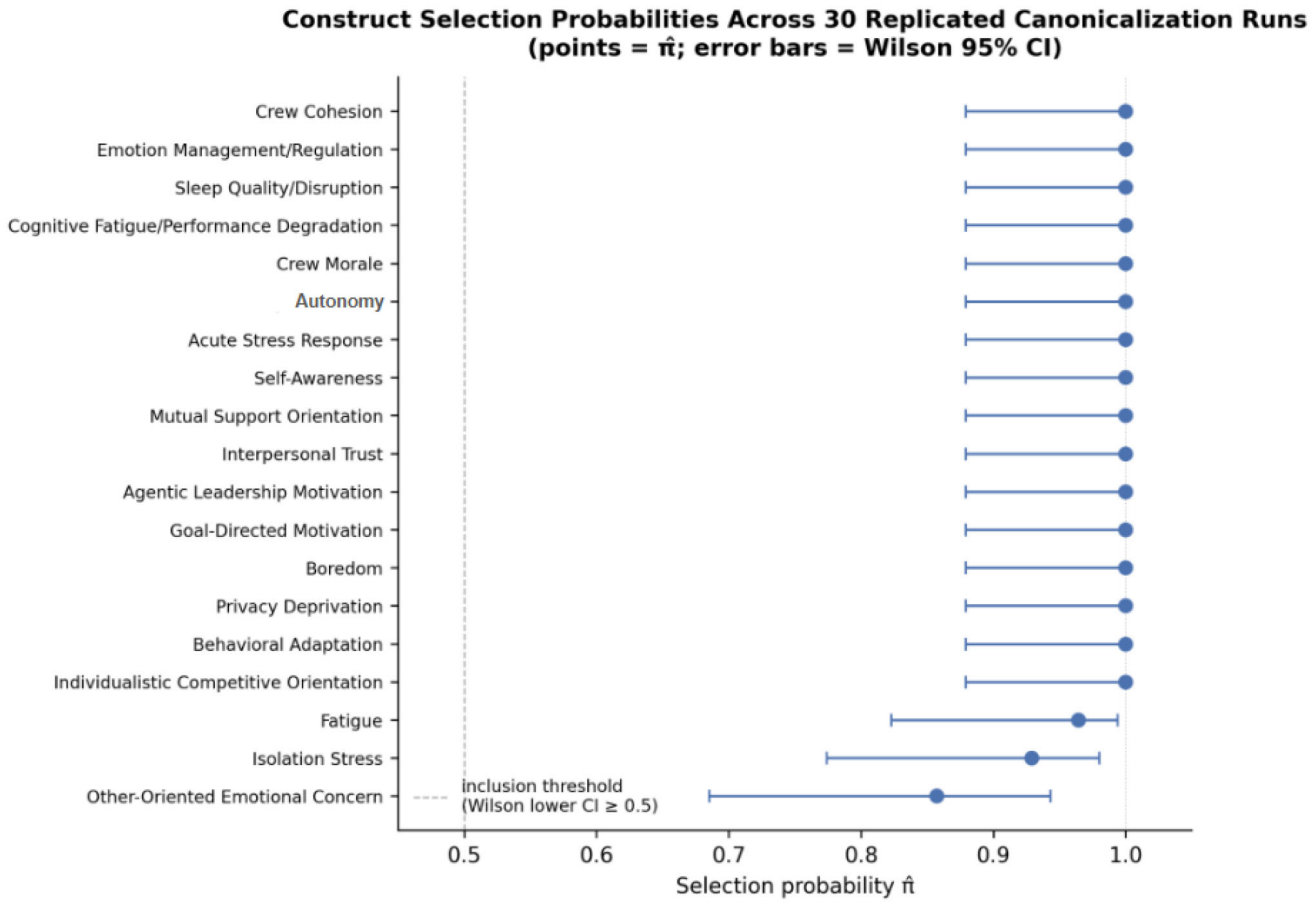
Construct selection probabilities across the 30 replicated canonicalization runs.

### 5.2 Stage 1 results: discovered constructs

Stage 1 analysis of the preprocessed MDRS crew log corpus, conducted through embedding, clustering, stability analysis, and expert validation, produced a set of 19 canonical psychological and cognitive constructs. The full construct inventory is presented in Table 1, ordered by passage frequency (the number of distinct retrieved log passages from which the construct was evidenced during the repeated-query extraction procedure). Passage frequency ranged from 26 for the most pervasive construct to 3 for the least frequent validated constructs and followed a Zipfian distribution consistent with the expected dominance of a small number of highly salient themes in naturalistic field text.

LLM-based construct identification showed strong agreement with both reference standards on the most prominent constructs, with performance degrading as passage frequency declined. Of the nineteen constructs evaluated, eleven (57.9%) received a “True” rating from both the NASA HRP HFBP Evidence reports and the human coder, indicating full convergent agreement. Notably, all four highest-frequency constructs [i.e., Crew Cohesion (26 passages), Emotion Management/Regulation (17), Sleep Quality/Disruption (14), and Cognitive Fatigue/Performance Degradation (11)] were rated True by both references, suggesting that the LLM identifies constructs most reliably when the source material provides a denser linguistic signal. Three constructs received converging “Partial” ratings from both references (i.e., Self-Awareness, Goal-Directed Motivation, and Boredom), and no construct was rated “None” by both, indicating that outright identification failures were rare.

Discrepant ratings between the two reference standards (*n* = 6) merit interpretive caution, as they reflect divergence between the gold standards themselves rather than unambiguous LLM error. In four cases (i.e., Crew Morale, Mutual Support Orientation, Behavioral Adaptation, Individualistic Competitive Orientation), the NASA evidence reports rated the LLM's identification as True while the human coder rated it Partial or None, suggesting that the LLM may over-anchor to the formal terminology used in NASA documentation at the expense of how constructs are operationalized in raw passages. The reverse pattern was observed in two cases (i.e., Autonomy, Goal-Directed Motivation), in which the human coder rated the LLM as True against a NASA rating of Partial. The mixed verdicts cluster among lower-frequency constructs (three to five passages), reinforcing the pattern that LLM reliability scales with construct prevalence in the source corpus.

The structural relationships among the validated constructs are visualized as a co-occurrence graph in Figure 3, rendered with a Kamada–Kawai layout and edges defined by chunk-level co-occurrence (minimum threshold of two). Several structural patterns emerge. *Crew cohesion* is the most prominent node by both passage frequency and degree, with co-occurrences spanning multiple domains. This suggests that crew members frequently reference cohesion when describing a wide range of other psychological and cognitive states. General *Fatigue* and *Cognitive Fatigue/Performance Degradation* function as a secondary hub. These nodes are densely connected to cognitive and interface-related constructs, forming a subcluster consistent with their shared phenomenology in the literature. *Emotion Management and Regulation* is situated within a social-motivational subcluster, indicating it is commonly co-articulated with teaming experiences and reflections on motivation. No isolated nodes were present at the specified threshold.

**Figure 3:**
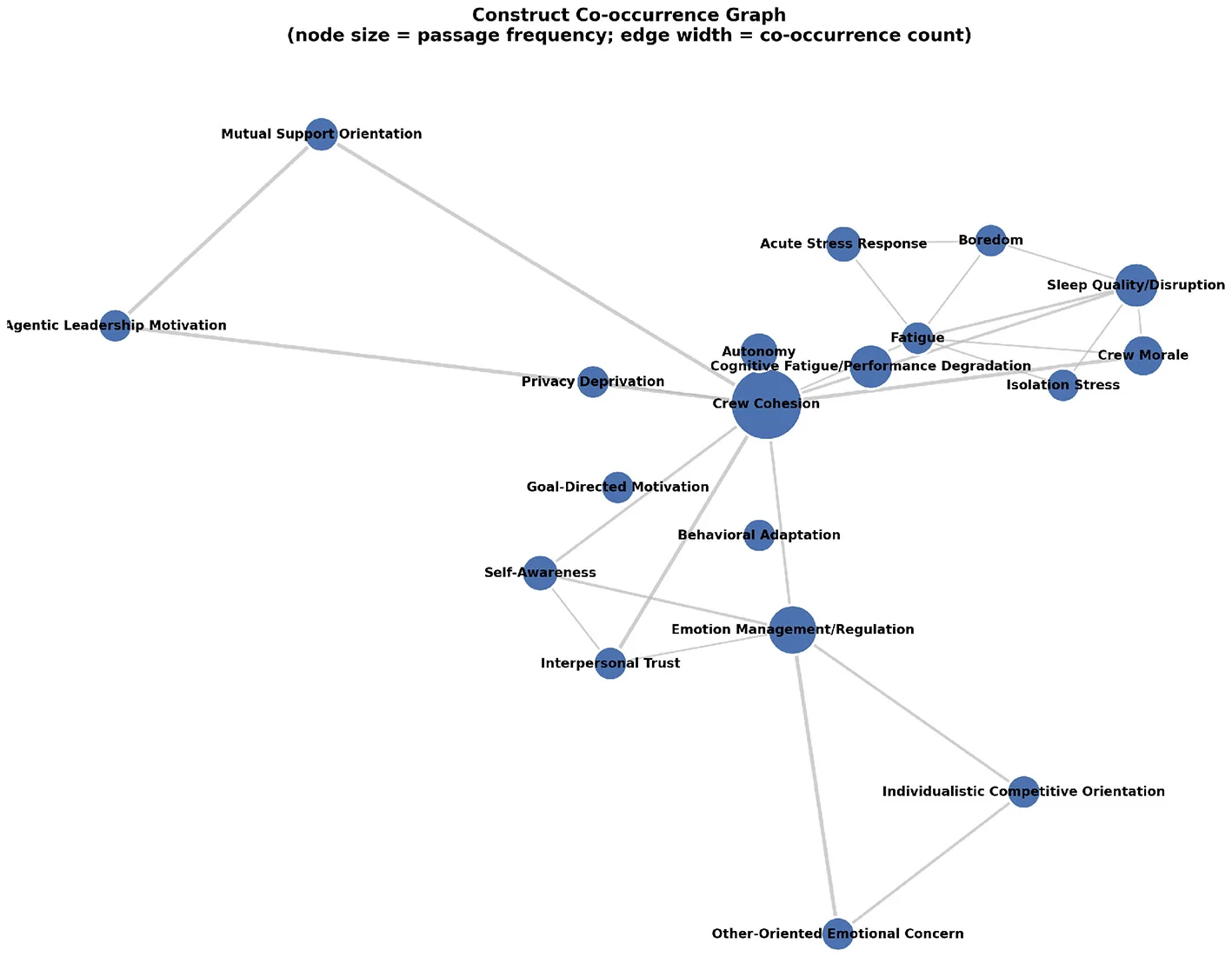
Co-occurrence graph of the structural relationships between the RAG-LLM-derived constructs.

### 5.3 Stage 1 results: RAG performance

To address our second research question about RAG performance, we assessed comprehensiveness, relevance, attribution correctness, and faithfulness to the prompt. The database of log entries was compared with what was available on the website, and it was determined that all 2,521 operational logs were fetched correctly, indicating that the system was comprehensive. For scope precision in relevance benchmarking, in a sample of 100 logs (Wilson margin ±10% at worst-case *p* = 0.5), 90% of evidence was relevant to the targets in the prompt. Additionally, with this sample, human raters confirmed that the LLM was in fact only retrieving data from the corpus. Further, in this sample, human raters agreed that 94% of the constructs identified faithfully answered the prompt.

### 5.4 Stage 2 results: evidence gap analysis

Stage 2 applied the literature retrieval pipeline and evidence rubric to each of the 19 validated constructs across four gap dimensions: ICE ecological validity, neural correlate identified, validated biosensor protocol, and BCI implementation ready. The complete construct-by-dimension evidence matrix is presented as a heatmap in Figure 4. Cell values range from 0 (red; no relevant evidence identified) to 3,236 (green; strong evidence), and rows are sorted so that the highest-gap constructs appear at the top of the matrix.

**Figure 4:**
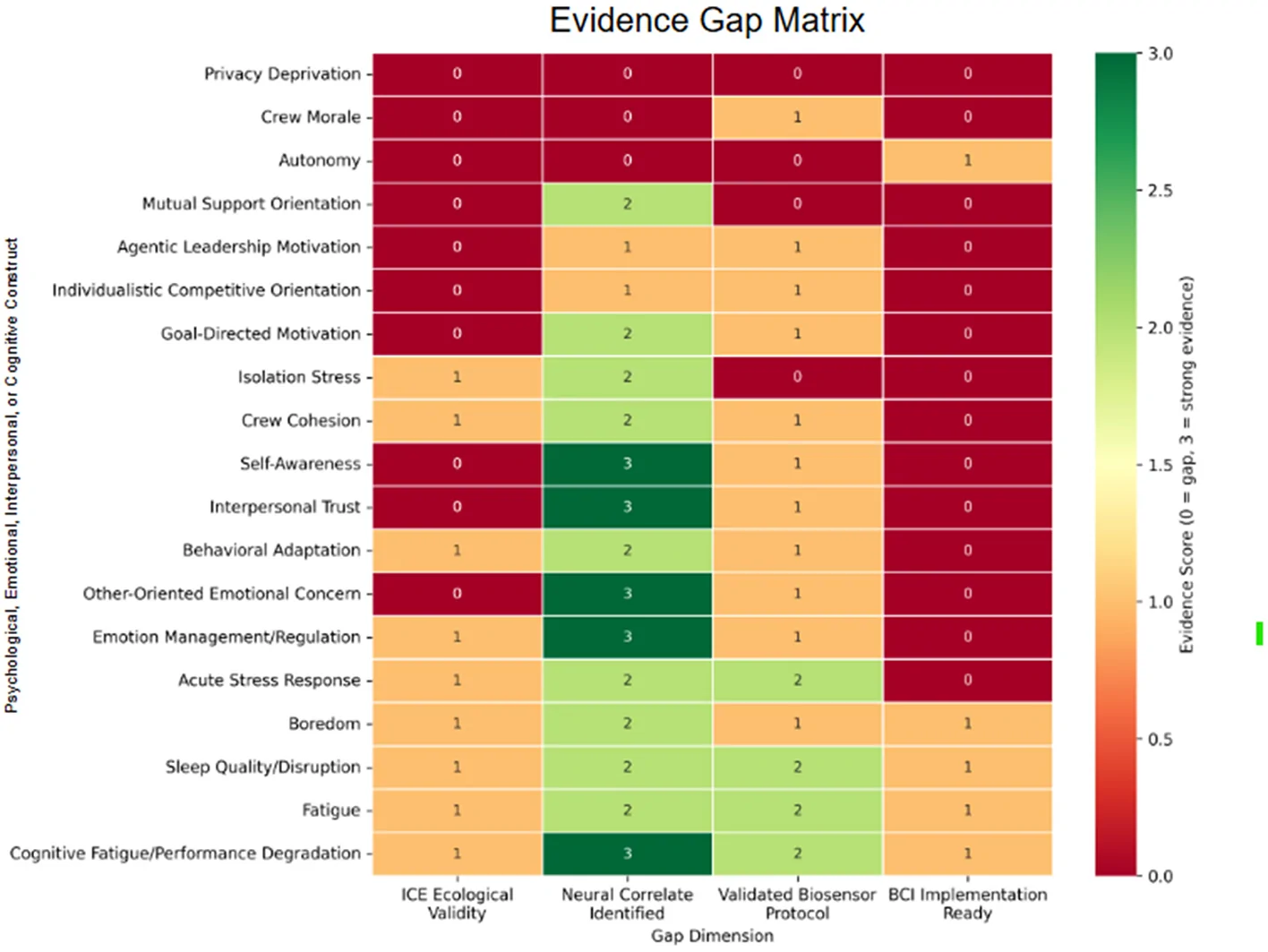
Neuroscience evidence gap matrix for ICE-environment mental-state constructs. Rows represent the 19 validated Stage 1 constructs; columns represent the four gap dimensions labeled in Stage 2. Cell values reflect the count of abstracts fetched after filtering and labeling; red indicates a larger gap, and darker green indicates a more mature evidence base.

Three patterns are immediately visible in Figure 4. First, the “BCI implementation ready” and “Validated Biosensor Protocol” columns show that overall fewer abstracts were labeled as supportive by the pipeline for constructs studied in these dimensions. Second, the “neural correlate identified” column shows the strongest evidence base overall, with most cognitive-affective constructs reflecting strong evidence counts—consistent with the well-known laboratory bias of the neuroimaging literature. Third, the social and affective constructs occupying the top rows of the matrix are underserved across every column, indicating that the phenomena most central to crew-authored field accounts are precisely those for which the translational pipeline from neuroscience to field deployment is least developed. These conclusions come with the caveat that the databases used by the pipeline may not have contained enough relevant literature to be represented accurately.

The top ten priority gaps are presented in Figure 5. Agentic Leadership Motivation and Crew Morale nearly tied for fewest abstracts across any of the four dimensions. Almost all of the top 10 represent the social states that are operationally salient in authentic field text and that the current literature does not yet support monitoring through any deployment-ready neurophysiological pathway. This skew reflects both the greater representation of those construct types in Stage 1 and the systematically weaker evidence base for social constructs relative to cognitive ones, especially in neuroimaging and BCI.

**Figure 5:**
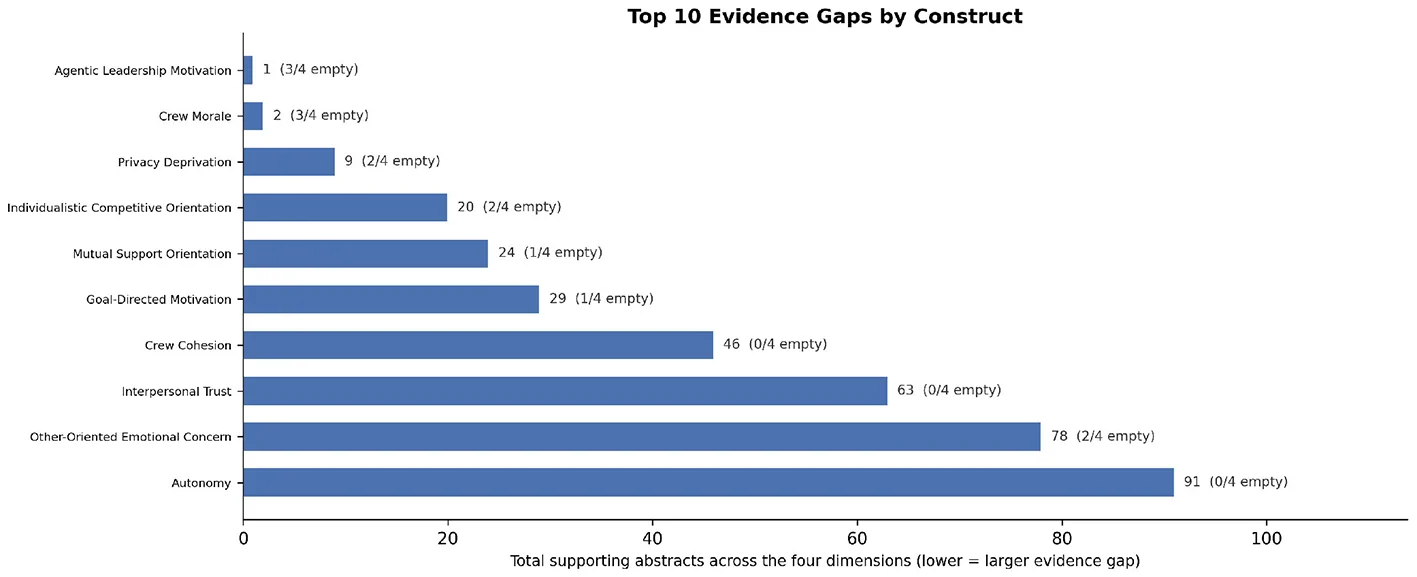
Top 10 research-priority gaps by construct, ranked by supporting abstract count, with each dimension-represented count in parentheses.

### 5.5 Stage 2 results: evaluation

The following describes the validation results. Citation cross-referencing flagged 3.6% of LLM-provided citations as not matching the retrieval database for the relevant construct. In terms of faithfulness, the LLM was explicitly citing from only the abstract corpus but the human–LLM agreement on the blinded sample of abstracts was initially only a pooled κ = 0.56. Following iterative prompt revision, this improved to pooled κ = 0.74. An expert reviewer flagged all matrix cells containing zero for re-evaluation using a manual search using the browser version of the databases. These replicated searches produced the same results indicating there were truly very few studies for the social constructs in the BCI Implementation dimension. The pipeline fetched 49,172 abstracts in 17 min. After filtering, PubMed contributed 19,396, Semantic Scholar contributed 11,237, and IEEE contributed 4,777. Out of that total, the set was further reduced to 33,149 after removing duplicate abstracts fetched by both databases or by different calls (PubMed retrieved 18,065 unique abstracts, Semantic Scholar retrieved 10,752 unique abstracts and IEEE retrieved 4,332 unique abstracts). In terms of comprehensiveness, we compared the total number of abstracts fetched in our final pass to the API's reported total one day later and found them equal. Our comparison to a two person team, results showed that in a literature search that took 2 h on each database site, 31 abstracts were evaluated. With this as a reference, the pipeline was more comprehensive than a human in less time. In terms of relevance, during filtering, 28% (*n* = 13, 762) were irrelevant and excluded at the API fetch stage. Using keyword searching of inclusion and exclusion criteria, the same research team deemed 13.9% of remaining abstracts were irrelevant to the construct the LLM assigned and removed them from the JSON before further processing, resulting in 28,541 abstracts evaluated by the LLM. The false-negative analysis indicated 99% of the filtered out abstracts were correctly removed indicating the deterministic filtering was highly effective.

## 6 Discussion

The two-stage pipeline produced an interpretable construct inventory and a quantitative evidence gap matrix from a corpus that has accumulated for more than two decades without being structured for research purposes. The three research questions guide this discussion: (1) What is the correspondence between what constructs field data reveal via RAG-LLM, via trained raters, and an expert taxonomy? (2) Is the RAG system accurate, relevant, comprehensive, and faithful? And (3) How well does the literature synthesis provide an accurate, relevant, comprehensive, and faithful summary of the state-of-the-art? This study then discusses the implications of the results and of using LLMs for research in this way, followed by limitations, future directions, and conclusions.

### 6.1 Correspondence between RAG-LLM, human raters, and expert taxonomy

LLM-based construct identification showed strong convergent agreement with both reference standards on high-frequency constructs, with all four most prevalent constructs (i.e., Crew Cohesion, Emotion Management/Regulation, Sleep Quality/Disruption, and Cognitive Fatigue/Performance Degradation) identified by both the NASA HRP HFBP Evidence reports and the human coder, and 57.9% of all nineteen constructs receiving full convergent agreement. All constructs discovered by the RAG-LLM corresponded in full or partially to the NASA HRP HFBP Evidence Book. A few constructs derived from the MDRS corpus were not frequently discussed in the NASA HRP HFBP Evidence, namely: acute stress response, self-awareness, goal-directed motivation, competitive orientation, and other-oriented emotional concern. Stress responses were discussed more generally in the Evidence and more often in the chronic sense, but the acuteness identified by the LLM may reflect the shorter duration of the MDRS missions—highlighting a possibly meaningful temporal confound when generalizing to longer-duration spaceflight. The other four psychological and psychosocial constructs were less represented in both reference standards but provide insight into additional competencies or personalities present in the spaceflight analog population. They also sit at the periphery with only one to two connections, suggesting these constructs may appear in distinctive narrative contexts whose passages do not heavily overlap with those evidencing the central hub constructs. Conversely, fatigue was identified as a densely connected node with known neural correlates and validated biosensor and BCI protocols. Combined with considerable literature retrieved about fatigue in ICE settings, this could indicate a low-risk research target that would fill gaps in ICE neuroergonomics.

Performance degraded for lower-frequency constructs, where mixed verdicts clustered and the two reference standards more often diverged in their assessments of the LLM than they agreed it had failed. This may indicate that apparent LLM error in this range often reflects definitional disagreement between the coding frameworks themselves rather than misidentification. It could also be a reflection of the sparseness of the constructs in the corpus, considering the most frequent construct only appeared 26 times within 2,521 logs. This pattern is consistent with the methodological argument that purely deductive coding constrains what qualitative data reveal (Braun and Clarke, 2006; Fereday and Muir-Cochrane, 2006). Although the present pipeline is computational rather than ethnographic, it operationalizes the same inductive logic that grounded theorists have long advocated (Charmaz, 2006), and at a corpus scale that manual analyses could not feasibly achieve. The high correspondence with the NASA HRP HFBP Evidence and independent human coding on a sampled subset lends weight to the claim that the LLM-derived inventory is not idiosyncratic to the model used. Overall, these results support scrutinous use of LLM-assisted construct coding for prominent, well-operationalized constructs while recommending human verification for rare or framework-specific constructs whose boundaries are themselves contested across reference standards.

### 6.2 The pipeline as a research instrument

A central concern for any LLM-mediated discovery and synthesis pipeline is whether the outputs can be trusted as research artifacts (Ji et al., 2023; Messeri and Crockett, 2024). This is the nature of research questions two and three. The validation results address this concern across several dimensions for both stages and answer our remaining research questions. Phase 1 comprehensiveness achieved a verified 100% with the MDRS archive. Phase 2 was built to be completely exhaustive of each database API. Both pipeline stages, however, demonstrated superior scalability, processing thousands of log entries relative to the double-digit throughput achieved via manual human analysis. Relevance, measured via human review on a per-construct sample, reached 90%, indicating the Stage 1 RAG-LLM was using highly relevant entries. Relevance was more varied in Stage 2 due to more noise sources. Faithfulness, defined as grounding outputs in retrieved corpus content rather than parametric knowledge, was confirmed by independent human verification on the same sample in both stages. In terms of accuracy, inter-rater agreement on the trained rater grounded theoretic canonicalization (κ = 0.76), on taxonomy correspondence (κ = 0.73), and on the blinded evidence gap abstract sample (pooled κ rising from 0.56 to 0.74 after iterative prompt revision) all exceeded conventional thresholds for substantial agreement. Citation cross-referencing flagged 3.6% of LLM-supplied references as missing from the retrieval database for the relevant construct, indicating that fabricated bibliographic entries, which constitute the most consequential form of hallucination for synthesis applications, occurred at very low rates with the prompt and retrieval regime used. Overall, both stages performed adequately on the evaluation metrics, with Stage 2 (which did not employ RAG) slightly worse.

Three implications follow from the construct pooling step, which aimed to create a reproducible list of constructs from the MDRS corpus despite LLM nondeterminism. First, the 19-construct inventory adopted as input to Stage 2 is reproducible to within the reported Wilson intervals under further replication of the pipeline, which addresses the principal concern about LLM nondeterminism in automated construct discovery. Second, the three sub-unanimous constructs are themselves diagnostic: they mark the boundaries of the construct space the pipeline can resolve at this corpus size and prompt configuration, and their identification is a useful output (they flag exactly the constructs where canonical-label choice would warrant human adjudication in a deployment setting). Third, the high mean pairwise Jaccard combined with the modest within-cluster label variation indicates that label variation, not construct selection, is the dominant source of run-to-run variance: the same constructs are being discovered each time, but their canonical phrasings drift across replications. Nevertheless, the final inventory of constructs was still reviewed and edited by a human, indicating the system should not be used unchecked.

Specific pipeline architectural choices supported these validation outcomes. Decoupling retrieval from inference by sweeping the corpus with broad seed queries and then performing per-chunk extraction over the deduplicated pool avoided the circularity of constructing per-construct retrieval queries before the construct vocabulary itself was known. Retrieval grounding via dense embeddings and vector indexing linked each construct identification back to specific source passages, satisfying the auditability requirement that distinguishes RAG from unconstrained generation (Lewis et al., 2020; Guu et al., 2020). The unconstrained extraction prompt, which deliberately withheld prior construct vocabularies, mitigated anchoring on parametric knowledge of psychological taxonomies. Followed by rigorous stability selection testing, these steps altogether reduced the risks of using LLMs.

These figures do not guarantee protection against subtler failure modes. The cross-referencing procedure detects fabricated titles but cannot detect cases in which the model cites a real retrieved paper while misrepresenting its findings. The κ values indicate adequate human-LLM agreement but show there are differences and do not explicitly exclude systematic bias shared between rater and model. Further, the human reviewer found a nontrivial percentage of articles that were irrelevant and that the automatic relevance filters did not identify. With those caveats in place, the cumulative evidence supports treating the pipeline output as a defensible *first-pass* synthesis suitable for downstream expert refinement rather than as an autonomous scientific product. This positioning aligns with the principles articulated for responsible LLM use in scientific contexts (Shah and Bender, 2022; Messeri and Crockett, 2024).

### 6.3 The joint picture: a translational gap

The most consequential scientific finding from the joint Stage 1 and Stage 2 output is structural rather than construct-specific. Crew-authored text most heavily emphasized social and affective phenomena, with the social domain populating the most prominent hub of the co-occurrence graph. Yet, the evidence counts reveal that these are precisely the phenomena the existing literature is least equipped to monitor. Critically, the evidence gap matrix shows that the deficit is not a uniform weakness but a two-dimensional gradient whose axes compound. The first axis is the construct domain: reading the matrix from its densely populated base toward its sparse top rows, evidence attenuates as one moves from physical and cognitive constructs (Fatigue and Sleep Quality/Disruption carry thousands of supporting abstracts across every dimension) to the social and motivational constructs (Crew Morale, Agentic Leadership Motivation, and Privacy Deprivation return single-digit or zero counts on most dimensions). The second axis is the translational stage. Within a construct, counts fall sharply as one moves from establishing that a neural correlate exists toward demonstrating a deployable biosensor or BCI protocol. This second gradient is the more diagnostic of the two, because it is visible even for constructs whose basic neuroscience is well-developed: Emotion Management/Regulation is supported by 3,122 neural-correlate abstracts but only 133 for a validated biosensor protocol and 167 for BCI implementation, and Self-Awareness collapses from 458 neural-correlate abstracts to 14 and 5, respectively. The bottleneck therefore likely sits at translation from laboratory correlate to field-deployable protocol, not at the absence of a neuroscientific basis. The two gradients intersect at the social and affective constructs most central to crew accounts, which occupy the least-developed construct domain and the least-developed translational stage simultaneously, driving their deployment readiness toward zero. This pattern is the empirical signature of a translational bottleneck long hypothesized in the neuroergonomics literature but rarely quantified across constructs simultaneously, and its most extreme expression—the near-absence of social-BCI evidence—was corroborated rather than produced by the pipeline: replicated browser searches matched the API totals, and the human review team independently confirmed that very few social-BCI studies exist.

Several established constraints in the literature account for the direction of this gradient. Until the rise of hyperscanning, methods that handle interacting brains, and more ecologically valid tasks, it has historically been challenging to tie neuroimaging metrics to social phenomena consistently (Maliske and Kanske, 2022). Many affective processes are, moreover, not consistently measurable by wearable neuroimaging devices because they engage deeper brain systems (e.g., fNIRS penetrates only around 3 cm and does not reach the amygdala). This may explain why abundant neural-correlate evidence for affective constructs does not carry through to the biosensor and BCI columns. Strangman et al. (2014) noted the difficulty of transferring laboratory-derived neural signatures to operational conditions, and Parasuraman (2003), and more recently Diarra et al. (2025) and Hamann et al. (2025) have documented that field neuroergonomic studies preferentially target constructs with established low-burden measurement pipelines. The evidence gap matrix quantifies this preferential pattern and, in doing so, distinguishes two qualitatively different research opportunities. Constructs with strong neural correlate counts but sparse biosensor and BCI evidence—the cognitive-affective mid-rows of the matrix—mark opportunities for protocol translation, where a demonstrated correlate awaits engineering into a deployable measurement. The social and motivational constructs at the top of the matrix, near-empty on every axis, instead mark where foundational neurophysiological work is still required before translation can begin.

The co-occurrence graph adds a complementary observation that may be useful for measurement design. Crew cohesion and fatigue were central nodes connected to many constructs and formed recognizable subclusters, but they anchor opposite ends of the readiness gradient: Fatigue is the most mature construct in the matrix, whereas the social hub around Crew Cohesion is among the least. Hub structures of this kind suggest measurement economies in which a single physiological monitoring device might productively serve more than one construct. Namely, instrumenting a well-characterized hub could work as an entry point into the under-served constructs it co-occurs with. Self-Awareness illustrates the principle: it is neurally well-understood (458 neural-correlate abstracts) yet sparsely evidenced for ICE ecological validity, biosensor protocols, and BCI, and it is connected deeply to Crew Cohesion, so investment in its field translation could plausibly extend to the social states it accompanies. Observations of this kind would be difficult to extract from any single construct's literature considered in isolation, but were uncovered here with the corpus-level structural analysis.

### 6.4 Implications for RAG-LLM use in field research contexts

The case study illustrates a practical role for RAG-LLM systems in operational field research. The role is not to generate scientific conclusions autonomously. Instead, it compresses otherwise prohibitive human effort into a tractable workflow whose outputs remain traceable. Manual review of more than two thousand log entries followed by manual evidence gap analysis across four evidence dimensions for the validated construct set would have required months of expert time. The pipeline reduced that effort to a workflow in which expert attention was concentrated where it adds the most value: validating constructs, synonyms, adjudicating sampled evidence dimension labeling, evaluating the system, and interpreting results. Han et al. (2024) and Ali et al. (2024) have argued that this redistribution of effort, rather than wholesale replacement of human reviewers, is the realistic near-term contribution of LLM-based synthesis to evidence aggregation. The findings reported here are consistent with that framing. Taken together, these findings support cautious use of LLM-assisted construct coding for high-prevalence, well-operationalized constructs. We recommend human verification for rare, context-specific, or framework-specific constructs where definitional boundaries between coding frameworks may themselves be a source of disagreement. The human reviewer could add additional options to the construct synonym list to increase the likelihood that the LLM identifies a log with described mental states.

A second implication concerns generalizability. The pipeline is corpus-agnostic at Stage 1 and construct-list-agnostic at Stage 2. Any operational environment that produces structured written records, including military after-action reviews, emergency response logs, submarine deployment journals, and financial trading-desk diaries, can in principle serve as input to Stage 1. Given the sparseness of constructs, however, we recommend screening new corpora. The evidence gap matrix dimensions could likewise be re-specified for any neurophysiological or non-neurophysiological measurement target of interest, but such changes should be subject to expert scrutiny. Given the limits discussed in Section 6.5, the pipeline is best understood as a starting place for research prioritization.

### 6.5 Limitations

Several features of the Stage 1 retrieval and construct discovery design constrain the interpretability of the resulting construct inventory and warrant explicit discussion. First, the corpus sweep retrieved chunks via cosine similarity to a fixed set of eight broad seed queries without enforcing balance across missions or contributing crew members; in principle, this allows the candidate construct pool to be over-weighted toward the writing style and recurring preoccupations of the most prolific log contributors. While the per-chunk extraction design (rather than per-call top-*k* batching) reduces the risk that any single contributor's voice dominates a single inference call, it does not formally bound per-author contribution to the deduplicated chunk pool. Future work should incorporate per-mission and per-author diversity constraints during retrieval. This may be implemented by enriching chunk metadata with mission identifiers, such as report-type parsed from log headers, and by post-filtering top-*k* retrievals to enforce minimum-coverage floors. Second, frequency-based filtering of candidate constructs (≥3 extraction events) treats each extraction event as evidentially equivalent, regardless of the surrounding chunk's information density or the LLM's stated confidence.

Stage 2 has its own limitations. Literature retrieval was restricted to abstracts and only three databases. Full-text retrieval was not implemented in this version of the pipeline, so evidence judgments may underweight findings reported in the body text but absent from the abstract. Publication-type filters, such as exclusions for letters, editorials, and conference abstracts, were not applied, allowing heterogeneity in the evidentiary quality of retrieved items. The four prioritization criteria, while operationally useful, compress ordinal evidence judgments and do not include more detail, such as whether a BCI protocol worked. Further, using only three article databases may have restricted the content retrieved, as potentially evidenced by the sparse BCI literature retrieved. These limits were likely exacerbated by the finding that there were indeed very few articles on social-BCI paradigms found by the human literature review team. Lastly, all queries were executed at a single point in time, and the resulting evidence cutoff date will become progressively stale as new neuroimaging work appears.

A broader limitation applies to both stages. The pipeline's outputs depend on the choice of LLM, embedding model, and prompting regime. Replicating with different model families would clarify how sensitive the construct inventory and evidence gap matrix are to those choices and is identified as a priority for follow-on work. Relatedly, the human validation steps draw on a small pool of experts, which constrains generalization of the inter-rater agreement statistics beyond the present sample.

### 6.6 Future work

Several extensions could strengthen the methodological contribution of the pipeline and broaden its scientific reach. Replicating with alternative LLM and embedding-model combinations would establish the robustness of the construct inventory and evidence gap matrix across model choices. Cross-model agreement statistics, computed on the same corpus and prompt regime, would either confirm or qualify the present results and address the model-dependence concern. Extending the literature retrieval pipeline to full-text rather than abstract-only inputs and testing different construct prioritization criteria could also be informative.

Beyond methodological refinement, several applications follow directly from the current results. Targeted protocol-development work on the maximum-gap social and affective constructs is the most direct response to the priority ordering surfaced by the evidence gap matrix. Such work would also test the pipeline's prospective validity in the strongest sense by establishing whether constructs flagged as priority gaps can be instrumented when methodological resources are directed at them. Conversely, the evidence gap matrix highlights the least risky avenues for ICE neuroergonomic research (i.e., fatigue has established neural correlates, biosensor protocols, and BCI implementations but is less well-studied in ICE).

Cross-corpus generalization and deeper exploration of the MDRS corpus are additional directions. Applying the same pipeline to non-MDRS operational corpora, including Antarctic overwintering logs, submarine deployment journals, military after-action reports, and emergency-response incident records, would test whether the construct inventory recovered here reflects features specific to the spaceflight analog context or generalizes across ICE and ICE-adjacent settings. Cross-corpus comparisons would also support the development of domain-general construct taxonomies that integrate bottom-up signals across multiple field environments. Last, longitudinal analyses within the MDRS corpus itself remain underexplored. Mission phase, season, and crew composition are candidate moderators of which constructs surface most prominently in crew-authored text. The archive's temporal granularity could support further tuning of this pipeline and analyses without additional data collection. Phase-conditioned construct trajectories could, in turn, inform the timing of measurement protocols in future field deployments.

## 7 Conclusion

This work presented a two-stage RAG-LLM pipeline for converting unstructured operational field text into a validated construct inventory and a quantitative neuroscience evidence gap matrix. Applied to more than two thousand crew logs from the Mars Desert Research Station, the pipeline surfaced 19 psychological, cognitive, and social constructs, of which the majority mapped partially or totally to existing expert taxonomies. The accompanying gap analysis identified three near-maximum-gap constructs, all in the social or affective domains, whose total supporting-abstract count across all four dimensions was under ten, and revealed a concentrated weakness in BCI implementation readiness across the construct space. Validation metrics support the position that the pipeline produces outputs credible enough to inform research priority decisions when paired with expert review. The contribution, namely the documentation of a translational gap concentrated in the social and affective domains most central to crew-authored field accounts, identifies a concrete gap for the neuroergonomics communities. Closing that gap will require sustained investment in laboratory neurophysiology of social and affective states, in field translation of the resulting protocols, and in the kind of bottom-up evidence aggregation that the pipeline reported here is designed to support.

## Data Availability

The raw data supporting the conclusions of this article will be made available by the authors, without undue reservation.
