# Supplementary material for: A two-stage RAG-LLM pipeline for roadmapping neuroscience research in operational environments
Source: Front Hum Neurosci. 2026 Oct 14;20:1884892. doi: 10.3389/fnhum.2026.1884892 (PMC13713852; doi:10.3389/fnhum.2026.1884892)
Supplement: Supplementary file 1 [file Supplementary_File_1.docx]

Supplemental Materials

1. Seed Queries

```
SEED_QUERIES = [
    'crew morale motivation and team dynamics',
    'stress fatigue cognitive performance and workload',
    'interpersonal conflict communication and leadership',
    'boredom isolation monotony and confinement',
    'decision making under uncertainty and time pressure',
    'emotional regulation anxiety and psychological wellbeing',
    'autonomy control and mission purpose',
    'sleep deprivation and physical discomfort',
]
```

1. Prompts

```
# ── Stage 1: Construct extraction ─────────────────────────────────────────
CONSTRUCT_EXTRACTION_SYSTEM = '''\
You are an expert in neuroergonomics, human factors, and ICE (isolated, confined, \
and extreme) environment psychology.

Your task is to extract MENTAL STATE CONSTRUCTS from field log excerpts written by \
crew members at the Mars Desert Research Station (MDRS) or similar analog environments.

A MENTAL STATE CONSTRUCT is a psychological or cognitive state that:
- Is internal to a crew member or the crew as a unit
- Could plausibly affect performance, resilience, or wellbeing
- Is distinct enough to be a research target (e.g., "fatigue", "crew cohesion", \
"autonomy frustration", "boredom")

Rules:
- Extract ONLY constructs EVIDENCED by specific language in the excerpt.
- Do NOT invent constructs not grounded in the text.
- Prefer precise psychological terms over vague ones.
- Return a JSON object ONLY — no preamble.

Output format:
{
  "constructs": [
    {
      "label": "short construct name (2-5 words)",
      "definition": "one-sentence definition as it appears in this context",
      "evidence_quote": "verbatim phrase from the text that supports this construct",
      "confidence": "high | medium | low"
    }
  ]
}'''

CONSTRUCT_EXTRACTION_USER = '''\
Extract mental state constructs from the following MDRS crew log excerpt:

<excerpt>
{chunk_text}
</excerpt>

Return valid JSON only.'''

# ── Stage 1: Clustering ────────────────────────────────────────────────────
CONSTRUCT_CLUSTERING_SYSTEM = '''\
You are a research psychologist specializing in taxonomy development for human \
performance in extreme environments.

Merge near-duplicate or overlapping constructs into canonical labels. \
Assign each to one domain: Cognitive, Affective, Social, Motivational, or Physical-Cognitive Interface. \
Rank by frequency.

Return JSON only:
{
  "constructs": [
    {
      "canonical_label": "...",
      "domain": "...",
      "merged_from": ["..."],
      "frequency": 0,
      "definition": "...",
      "representative_quote": "..."
    }
  ]
}'''

CONSTRUCT_CLUSTERING_USER = '''\
Here are {n_constructs} raw constructs extracted from {n_chunks} log chunks. \
Cluster and canonicalize them:

<raw_constructs>
{constructs_json}
</raw_constructs>

Return valid JSON only.'''

# ── Stage 2: Source comparison ─────────────────────────────────────────────
SOURCE_COMPARISON_SYSTEM = '''\
You are a research librarian evaluating two scientific literature databases \
for neuroscience gap analysis. Compare PubMed vs Semantic Scholar results \
for the same construct. Return JSON only:
{
  "construct": "...",
  "coverage_winner": "pubmed | semanticscholar | tie",
  "coverage_reasoning": "...",
  "relevance_winner": "pubmed | semanticscholar | tie",
  "relevance_reasoning": "...",
  "unique_to_pubmed": ["..."],
  "unique_to_semanticscholar": ["..."],
  "recommendation": "..."
}'''

SOURCE_COMPARISON_USER = '''\
CONSTRUCT: {construct_label}
PUBMED GAP REPORT:
{pubmed_report}
SEMANTIC SCHOLAR GAP REPORT:
{s2_report}

Return your comparison as JSON only.'''
```

print('✓ Prompts loaded')

2. Pairwise Cosine-Distance Distribution Across Pooled Constructs

Distribution of pairwise cosine distances among construct labels pooled across all MDRS log chunks (bin width = 0.01). The distribution is bimodal: a small low-distance mode (peak around 0.01), which plausibly reflects near-duplicate or paraphrased labels, is separated by a sparse trough (~0.07-0.10) from the main mass of pairs (mode: 0.23), presumably distinct constructs. Dashed lines mark candidate merge thresholds: 0.08 falls near the antimode, whereas 0.05 cuts into the low-distance mode and 0.12 reaches the rising flank of the main mode. Because pair counts scale quadratically with cluster size, the low-distance mode overstates the number of redundant constructs.

3. Construct Synonyms (based on interchanged terminology in the literature)

| Crew Cohesion | team cohesion, group cohesion, social cohesion, unit cohesion, interpersonal bonding |
| --- | --- |
| Emotion Management/Regulation | emotion regulation, affect regulation, emotional coping, emotional labor, self-regulation of affect |
| Sleep Quality/Disruption | sleep quality, sleep disturbance, sleep disruption, subjective sleep, sleep impairment, insomnia symptoms |
| Cognitive Fatigue/Performance Degradation | cognitive fatigue, mental fatigue, cognitive performance decrement, neurobehavioral performance decline, cognitive impairment |
| Crew Morale | group morale, team morale, collective affect, positive group affect, group emotional climate |
| Autonomy Frustration | autonomy thwarting, perceived loss of control, restricted agency, psychological need frustration, self-determination thwarting |
| Acute Stress Response | acute stress reaction, physiological stress response, cortisol response, sympathetic stress activation, HPA axis activation |
| Self-Awareness | emotional awareness, interoceptive awareness, self-reflection, emotional clarity, mindful self-awareness, introspection |
| Interpersonal Trust | team trust, psychological safety, relational trust, group trust, trust formation |
| Mutual Support Orientation | social support, peer support, perceived team support, communal coping, prosocial team behavior |
| Agentic Leadership Motivation | agentic leadership, task-oriented leadership, goal-directed leadership, instrumental leadership, achievement motivation in leaders |
| Goal-Directed Motivation | goal commitment, achievement motivation, task motivation, mission engagement, purposeful engagement |
| Boredom | state boredom, monotony, understimulation, task disengagement, tedium |
| Privacy Deprivation | lack of privacy, crowding stress, loss of personal space, confinement stress, privacy regulation |
| Behavioral Adaptation | behavioral adjustment, psychological adaptation, acclimatization, behavioral acclimation, adaptive behavior or thinking |
| Individualistic Competitive Orientation | competitive orientation, individualism, competitiveness, interpersonal rivalry, achievement competitiveness |
| Fatigue | physical fatigue, general fatigue, exhaustion, tiredness, operational fatigue |
| Isolation Stress | social isolation, confinement stress, loneliness, ICE environment stress, psychosocial isolation |
| Other-Oriented Emotional Concern | empathic concern, communal orientation, prosocial concern, other-focused empathy, interpersonal sensitivity |

4. NASA HRP HFBP construct correspondence breakdown by appearance in each evidence report.

| **Construct** | **BMed 2025** | **HSIA 2023** | **Team 2022** | **Sleep 2016** | **Dynamic Loads** |
| --- | --- | --- | --- | --- | --- |
| Crew Cohesion | 12 | 4 | 138 | 2 | 0 |
| Emotion Management/Regulation | 28 | 0 | 5 | 2 | 0 |
| Sleep Quality/Disruption | 10 | 40 | 28 | 145 | 0 |
| Cognitive Fatigue or Performance or Degradation | 73 | 8 | 37 | 34 | 0 |
| Crew Morale | 10 | 3 | 7 | 1 | 0 |
| Autonomy | 29 | 22 | 116 | 2 | 0 |
| Acute Stress Response | 6 | 1 | 2 | 2 | 0 |
| Self-Awareness | 3 | 1 | 0 | 0 | 0 |
| Interpersonal Trust | 6 | 0 | 10 | 0 | 0 |
| Mutual Support Orientation | 36 | 0 | 27 | 0 | 0 |
| Agentic Leadership Motivation | 6 | 1 | 0 | 1 | 0 |
| Goal-Directed Motivation | 1 | 1 | 0 | 0 | 0 |
| Boredom | 15 | 4 | 2 | 2 | 0 |
| Privacy Deprivation | 12 | 5 | 1 | 0 | 0 |
| Behavioral Adaptation | 30 | 0 | 1 | 2 | 0 |
| Individualistic Competitive Orientation | 1 | 0 | 1 | 0 | 0 |
| Fatigue | 10 | 26 | 1 | 107 | 1 |
| Isolation Stress | 7 | 15 | 4 | 1 | 0 |
| Other-Oriented Emotional Concern | 1 | 1 | 1 | 1 | 0 |
